# Fluorinated Iron Phthalocyanine–rGO Hybrid Cathodes for Fast and Stable Aqueous Zinc Batteries

**DOI:** 10.1021/acsomega.6c05388

**Published:** 2026-08-24

**Authors:** Andres E. Araujo-Correa, Yamna D. Acosta-Tejada, Lexis N. Feliz-Hernandez, Wentao Hou, Laura Catalina Cruz, Lisandro Cunci, Karilys González-Nieves, Xianyong Wu, Dalice M. Piñero Cruz

**Affiliations:** † University of Puerto Rico-Rio Piedras, San Juan 00925, Puerto Rico; ‡ University of Puerto Rico-Carolina, Carolina 00984, Puerto Rico

## Abstract

Aqueous zinc batteries have become an attractive alternative for energy storage due to their low cost, safety, and low toxicity compared to traditional lithium-ion batteries, which suffer from safety concerns. However, they are limited by the lack of high-performance, stable cathodes. Herein, we report the electrochemical study of iron phthalocyanine-reduced graphene oxide hybrid materials as cathodes for aqueous zinc batteries. The prepared materials had a voltage of 1.2 V and displayed high cyclic stability with a capacity retention of over 70% after 2500 cycles and a high-rate performance up to 2000 mA/g, which represents an improvement compared to bare iron phthalocyanine, highlighting the positive effect of the incorporation of reduced graphene oxide to prepare a hybrid. Furthermore, incorporating fluorine into the phthalocyanine ring led to improved battery performance, which highlights the positive effect of tuning the phthalocyanine ring for the design of high-performance phthalocyanine-based cathodes for aqueous zinc-ion batteries.

## Graphical abstract

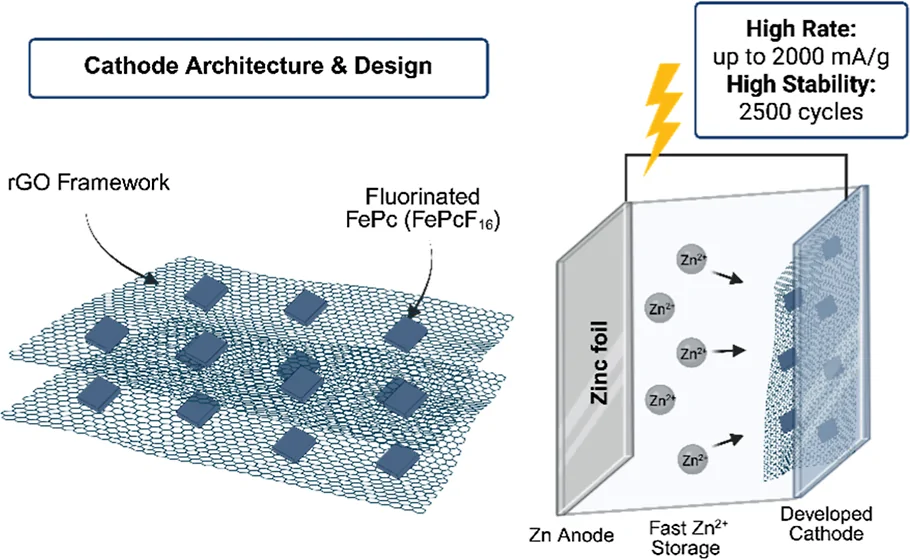

## Introduction

Lithium-ion batteries have long been the industry standard for commercial batteries.^1^ However, due to their environmental impact, scarcity, and theoretical capacity (278 mAh/g),^2^ they are unable to meet growing energy demands. Thus, researchers have focused on the development of next generation batteries, in which aqueous batteries have garnered increased attention due to their improved safety, low environmental impact, higher ionic conductivity, and low cost.^3^ Among them, aqueous zinc-based batteries are an attractive alternative due to zinc’s theoretical capacity (820 mAh/g), low cost, and low toxicity of zinc.^4^

Cathode materials have been categorized as either inorganic or organic materials. Inorganic cathodes such as vanadium oxide,^5^ manganese oxide,^6^ and Prussian blue analogues^7^ often provide high capacities and structural stability, whereas organic cathodes are more cost-effective, tunable, and flexible, though they typically exhibit lower electrical conductivity and stability compared to inorganic materials.^8–11^

Because of this, metal–organic materials, which feature the redox properties of the transition metals as well as the structural tunability of organic cathodes, have gathered increased attention in recent years.^12^ Among these types of material, Metal Phthalocyanines (MPc), the synthetic analogues of porphyrins, offer an attractive alternative to traditional cathodes due to their chemical and thermal stability, extensive conjugated π network, and electronic properties. These properties can be tuned by peripheral or nonperipheral substitution and the change of the central metal atom.^13,14^

MPcs have been used in a wide range of applications, such as sensors,^15,16^ optics,^17^ catalysis,^18–21^ and batteries.^22–26^ However, in standard aqueous zinc batteries, there are limited reports of their use. Yan et al. reported a phthalocyanine-based polymer that achieved a specific capacity of 269 mAh/g and showed good cyclic performance up to 1000 cycles.^27^ Additionally, Hwang et al. prepared a flexible MPc Zinc battery using ZnSO_4_ as an electrolyte, which investigated different metal centers and determined that iron phthalocyanine (FePc) performed best up to 100 cycles.^28^ Previous research efforts from our group found that utilizing highly concentrated salts in water-in-salt electrolytes prevents MPc’s dissolution in the aqueous electrolyte and increases the cyclic performance of the unsubstituted MPcs.^29^

However, more research is needed to increase the capacity of MPcs. To achieve this, changing the phthalocyanine ring with substituents such as F, NO_2_, or OH, which alter the material’s electronic properties and increase conductivity, will improve battery performance.^25^ Furthermore, due to their extensive π network, incorporating them into carbon-based supports, such as carbon nanotubes, carbon nanorods, carbon nano straws, and reduced graphene oxide (rGO), via π–π stacking allows for the preparation of hybrid materials with synergistic effects for energy applications.^30^ Liu et al.^31^ and Wu et al.^32^ reported the incorporation of FePc into carbon nanorod and nano straw hybrids, which outperformed a benchmark platinum and ruthenium catalyst in zinc-air batteries, highlighting the benefit of incorporating FePc into carbon-based supports. The benefit of this type of hybrids is further shown by Qi et al., who designed a CoPc­(NO_2_)_4_/rGO hybrid, which showed a synergistic effect between the MPc and rGO, increasing the capacity in a supercapacitor.^33^ rGO, specifically, has been observed to increase the performance of vanadium-based cathodes in aqueous zinc batteries,^5^ showcasing the potential benefits of incorporating rGO into iron phthalocyanines.

Herein, we report the preparation of FePc-rGO and FePcF_16_-rGO hybrids and their electrochemical characterization as cathodes for aqueous zinc batteries, in which both materials exhibit a long cyclic performance of 2500 cycles and a rate capability of up to 2000 mA/g. Additionally, this work highlights the influence of the substituents in the phthalocyanine range on battery performance.

## Experimental Section

### Materials

All chemicals and reagents were commercially purchased and used without further purification. *N*,*N*-dimethylformamide (DMF), acetone, ethanol, hexane, *N*-methyl-2-pyrrolidone (NMP), polyvinylidene fluoride (PVDF), and zinc chloride (ZnCl_2_) were purchased from Sigma-Aldrich. Iron­(II) acetate (Fe­(OAc)_2_), phthalonitrile (Pn), and tetrafluorophthalonitrile (PnF_4_) were obtained from Fisher Scientific Co. Polytetrafluoroethylene (PTFE) was purchased from Landt Company. Zinc foil was purchased from Amazon and had a thickness of 0.1 mm. Carbon fiber current collectors were purchased from the Fuel Cell store and had a thickness of 0.37 mm and a diameter of 1 cm.

### Characterization

Fourier transform infrared spectroscopy (FT-IR) was performed using a Bruker Alpha II. UV–vis absorption spectra were recorded utilizing a Shimadzu UV-2600I. Raman spectra were obtained using a 532 nm laser. Scanning electron microscopy (SEM) and energy-dispersive X-ray spectroscopy (EDS) were performed with a Phenom Pharos G2 from Thermo Fisher Scientific Corporation. Nuclear magnetic resonance (NMR) was performed with a Bruker 500 Hz. Cyclic voltammetry (CV) and electrochemical impedance spectroscopy were done using a Biologic SP-150 potentiostat. Finally, rate performance, cyclic performance, and galvanostatic charge and discharge were conducted on a Landt battery cycler (CT3002 AU). X-ray diffraction (XRD) was performed utilizing a XRD Rigaku MiniFlex with a Cu Kα radiation source and a wavelength of 1.5418 Å. Ex situ XRD were collected utilizing a Rigaku SuperNova HyPix300 Microdiffractometer with a multilayer mirror-monochromated Cu Kα radiation source and a wavelength of 1.5418 Å.

### Synthesis of Iron Phthalocyanine (FePc)

The synthesis was performed following the procedure reported by Denekamp et al.^34^ In a Teflon reactor, 500 mg of Pn and Fe­(OAc)_2_ were added, and the reactor was sealed under an inert atmosphere. Afterward, it was placed in an oven at 250 °C for 3 h. The crude solid was then washed with water and hexane and filtered to obtain FePc as a black powder. The material was characterized by FT-IR, Raman spectroscopy, XRD, and UV–vis.

### Synthesis of Hexadecafluorinated Iron Phthalocyanine (FePcF_16_)

The synthesis was performed following the procedure reported by Denekamp et al.^34^ In a Teflon reactor, 500 mg of PnF_4_ and Fe­(OAc)_2_ were added and sealed under an inert atmosphere. Afterward, it was placed in an oven at 250 °C for 3 h. The crude solid was then washed with water and hexane. It was followed by Soxhlet extraction using acetone as a solvent to obtain the desired product as a black powder after evaporating the solvent under vacuum. The material was characterized by FT-IR, Raman spectroscopy, XRD, UV–vis, and ^19^F NMR.

### Synthesis of Reduced Graphene Oxide

rGO was obtained from graphite following a modified Hummers method to obtain GO.^30^ Afterward, 500 mg of GO was added to a 100 mL Schlenk tube and dispersed in dry DMF via sonication for 2 h. Following this, hydrazine and ammonia–water were added to the solution, which was then placed in an oil bath at 100 °C for 24 h under nitrogen. The reaction was then filtered and washed with DMF, ethanol, and acetone to obtain rGO as a black powder.

### Synthesis of FePc-rGO and the FePcF_16_–rGO Hybrid

100 mg of rGO was added in a 100 mL Schlenk tube and was dispersed in dry DMF for 2 h before the dropwise addition of 200 mg of FePc or FePcF_16_, which was previously dissolved in DMF, and sonicated for an additional 2 h. This was followed by stirring at room temperature for 24 h. The solution was then filtered and washed with DMF, ethanol, and acetone to obtain the desired hybrid.^33^

### Electrode Preparation

To prepare the cathode, a slurry was prepared using 14 mg of hybrid material, 4 mg of Ketjen black carbon, and 2 mg of PVDF as a binder for a ratio of 7:2:1. NMP was added to obtain the desired consistency of the homogeneous slurry, which was then cast on carbon fiber (thickness: 0.37 mm; diameter: 1.0 cm) and dried under vacuum at 60 °C for 24 h. The prepared electrodes had an average mass loading of 1–1.2 mg/cm^2^ based on the total mass of the hybrid. Electrodes for ex-situ XRD were prepared as a self-standing film utilizing 60% PTFE as a binder and water as the solvent in the same 7:2:1 ratio as the standard electrodes. The dimensions of the electrode were 5.15 mm × 2.36 mm for a total mass loading of 4.78 mg/cm^2^.

### Battery Assembly

The prepared electrode was then used in aqueous zinc batteries with a Zn film as the anode, a commercial glass fiber separator, ZnCl_2_ 30 M as the electrolyte, and the FePc-rGO electrode as the cathode, assembled in a Swagelok cell configuration.

## Results and Discussion

### Synthesis and Characterization of the MPc–rGO Hybrid

FePc–rGO and FePcF_16_–rGO hybrids were prepared following previously reported procedures, as shown in Scheme 1.^33^

**Scheme 1:**
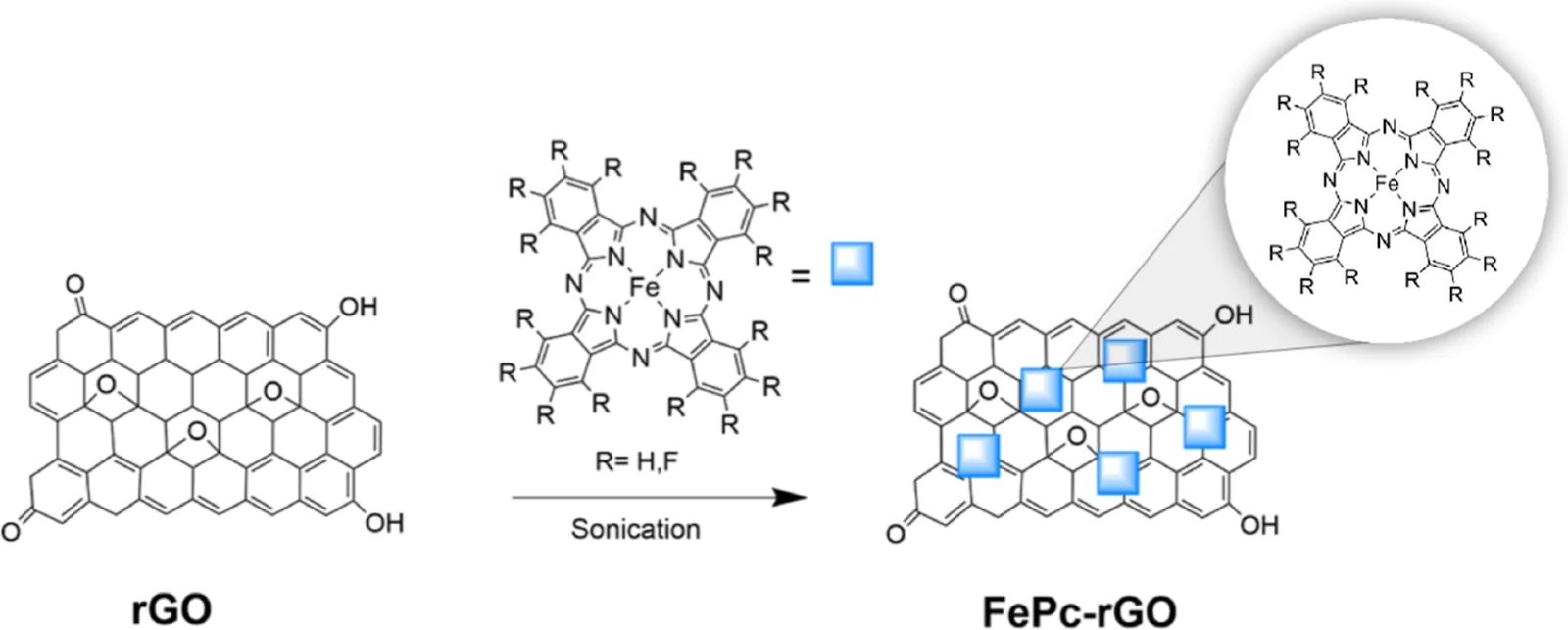
Reaction Scheme for the Preparation of the FePc–rGO Hybrid

UV–vis Spectroscopy is widely used to characterize MPcs since they exhibit two distinct peaks corresponding to π–π* interactions at around 600 nm (Q-band) and 300 nm (B band).^35^ Figure 1a shows the Q and B bands of the FePc, FePcF_16_, rGO, and their respective hybrids. The FePc–rGO hybrid maintained both peaks with a red shift, going from 654 to 657 nm and 321 to 329 nm, which can be attributed to the interactions between the FePc and rGO. Additionally, pristine FePcF_16_ had a λ_max_ of 661 and 317 nm. The difference in λ_max_ compared to FePc can be explained by the presence of fluorine atoms, which reduce the energy barrier for the π–π* electronic transition.^36^ In the same way as FePc, the incorporation of FePcF_16_ into the rGO caused a red shift of the Q-band from 661 to 671 nm.^30^

**Figure 1:**
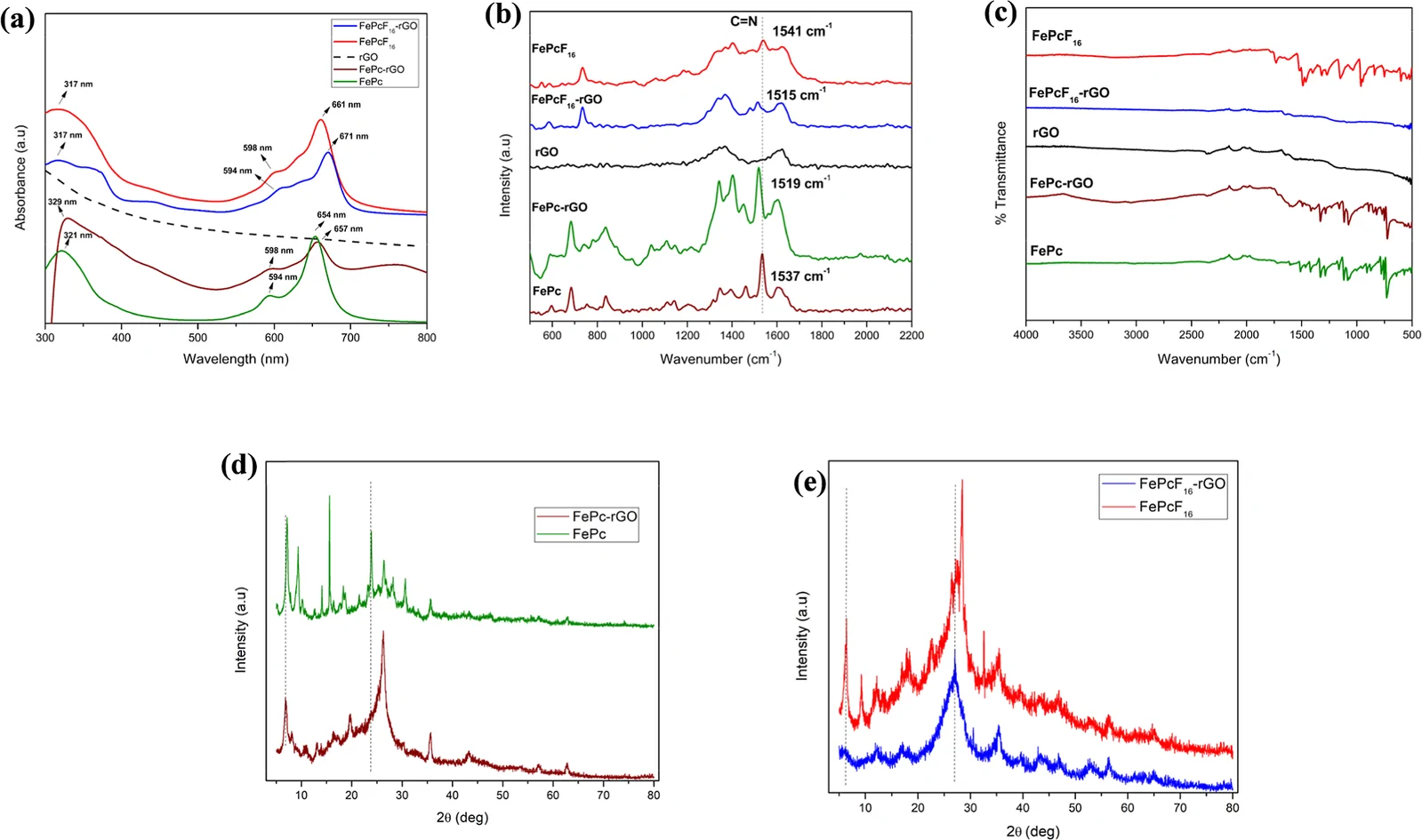
(a) UV–vis spectroscopy of FePc, rGO, FePc–rGO, FePcF_16_, and FePcF_16_–rGO in DMSO. (b) Raman Spectroscopy of FePc, rGO, FePc–rGO, FePcF_16_, and FePcF_16_–rGO. (c) FT-IR of FePc, rGO, FePc–rGO, FePcF_16_, and FePcF_16_–rGO. (d) XRD of FePc and FePc–rGO. (e) XRD of FePcF_16_ and FePcF_16_–rGO.

Raman spectroscopy was performed to further characterize the material, and Figure 1b illustrates the spectra of FePc, FePcF_16_, rGO, and the prepared hybrids. The FePc–rGO hybrid showed a shift in the peak at 1537 cm^–1^, which corresponds to the C=N stretching compared to pristine FePc at 1519 cm^–1^, which once again suggests electronic interaction between FePc and rGO in the hybrid material.^37^ The FePcF_16_–rGO hybrid, just like the FePc–rGO hybrid, had a shift in the wavenumber of the peak associated with the stretching of C=N from 1541 cm^–1^ to 1515 cm^–1^ due to the electronic interactions of FePcF_16_ with the rGO framework.^37^ Finally, FT-IR shows that the peaks corresponding to the MPc are maintained in the prepared hybrids at 1480 cm^–1^, 1100 cm^–1^, and 800 cm^–1^, as seen in Figure 1c.

Figure 1d shows the XRD patterns of FePc and the FePc–rGO hybrids; the diffraction peaks of FePc are consistent with a β-phase of the FePc with prominent diffraction peaks at 7.2°, 9.4°, 15.3°, and 23.9°.^38^ The FePc-rGO features shifted peaks at 6.8° and 26.2°, which confirms the loading of FePc into the rGO substrate. Finally, Figure 1e displays the XRD pattern of FePcF_16_, which features a prominent peak at 28.4°, which is consistent with a β-phase of the FePcF_16_.^38^ The FePcF_16_–rGO hybrid had the peak shifted to 27.0°, indicating the loading of FePcF_16_ into the rGO.

Furthermore, Figures 2 and 3 feature SEM images of both FePc–rGO and FePcF_16_–rGO hybrids, which display an amorphous morphology. EDS analysis (Tables S1 and S2 and Figures S4 and S5) confirms the incorporation of the MPc into the rGO, in which the composition for FePc–rGO, in weight %, was 53.85% C, 19.78% N, 11.99% O, and 14.39% Fe. In the case of the FePcF_16_–rGO hybrid, the weight % compositions were 55.75% C, 9.48% N, 13.71% O, 14.01% F, and 7.06% Fe. Elemental mapping further shows the homogeneous distribution of each element in the hybrids, confirming the successful preparation of the desired materials.

**Figure 2:**
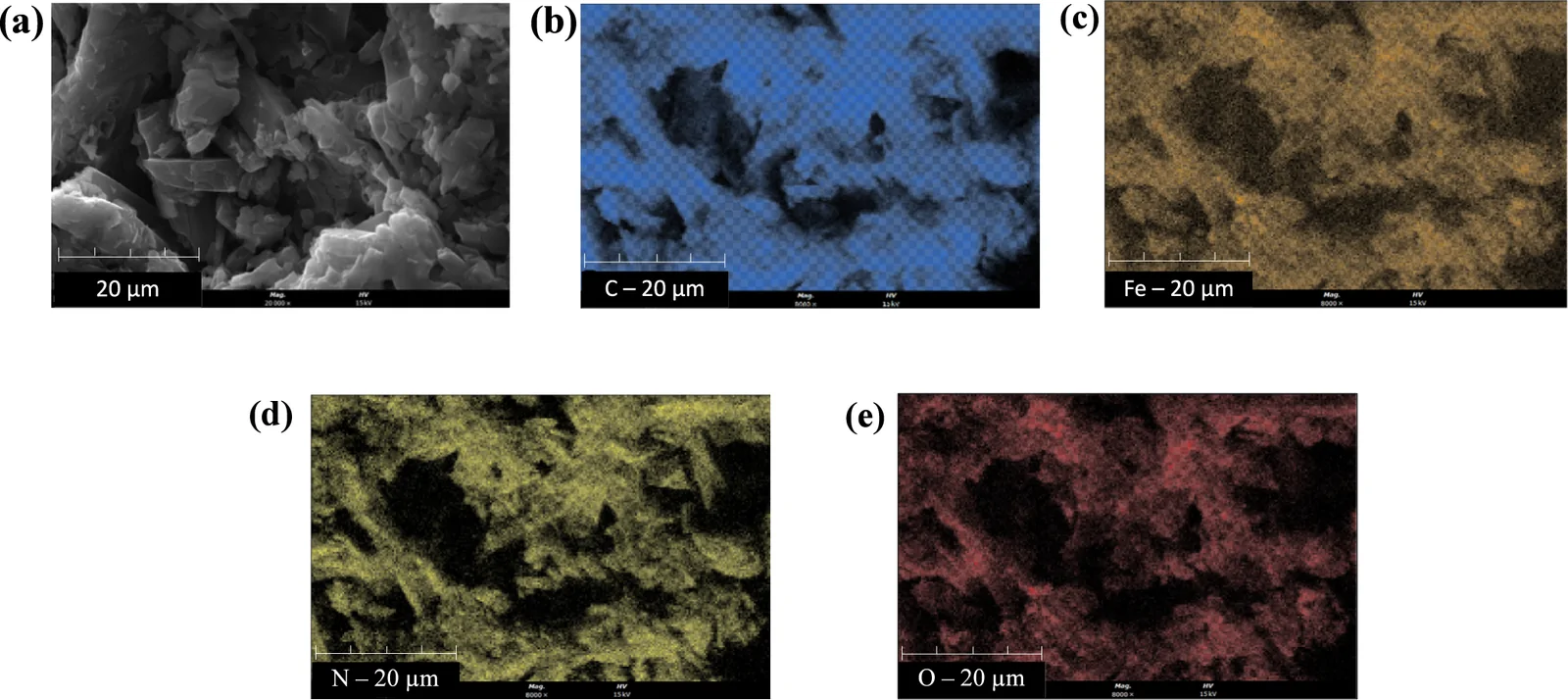
(a) SEM of the FePc–rGO hybrid. (b) Elemental mapping of C. (c) Elemental mapping of Fe. (d) Elemental mapping of N. (e) Elemental mapping of O.

**Figure 3:**
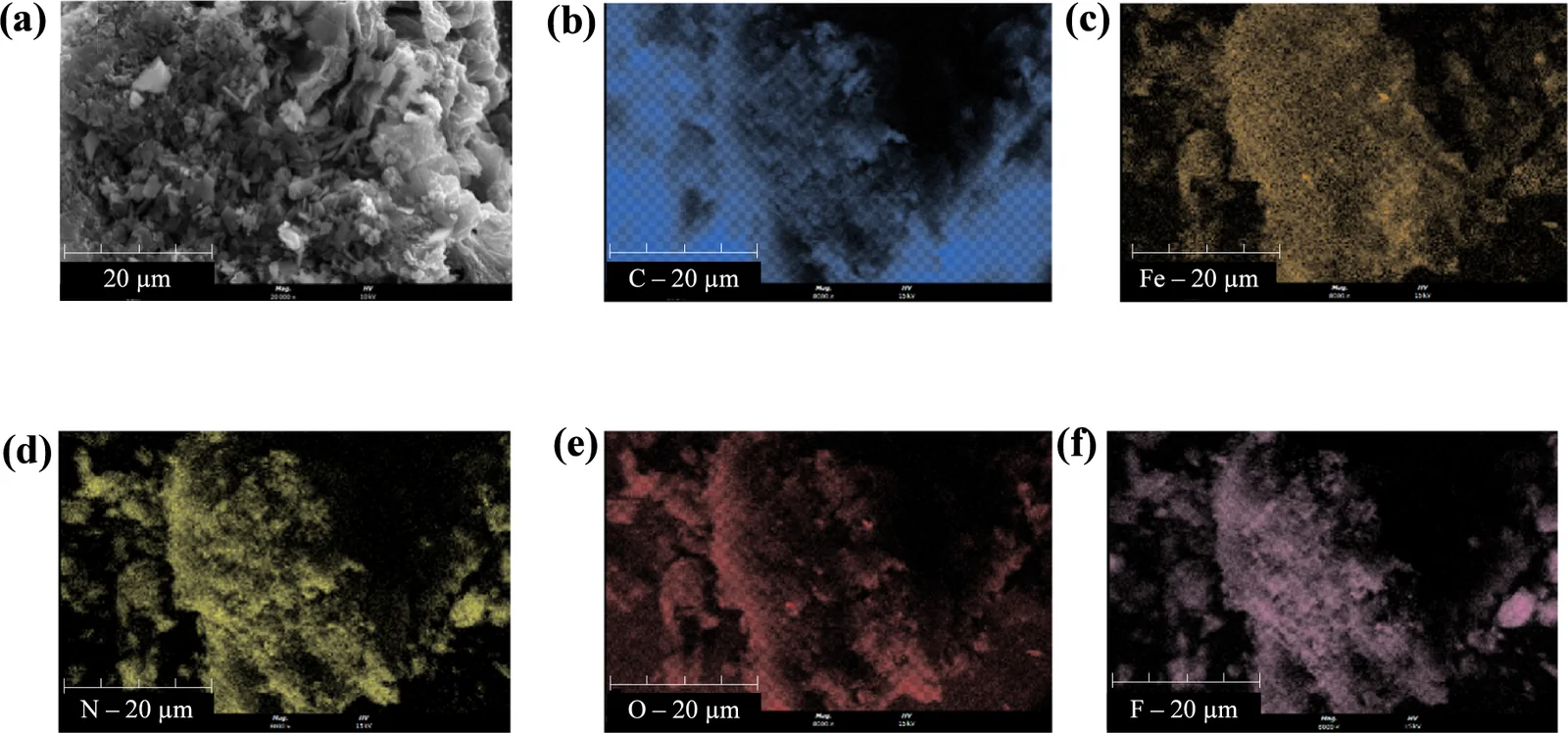
(a) SEM of the FePcF_16_–rGO hybrid. (b) Elemental mapping of C. (c) Elemental mapping of Fe. (d) Elemental mapping of N. (e) Elemental mapping of O. (f) Elemental mapping of F.

### Electrochemical Characterization of MPc-Hybrid Cathodes

FePc–rGO and FePcF_16_–rGO hybrids were tested as cathodes in aqueous zinc batteries. Figure 4a,b show the corresponding galvanostatic charge–discharge (GCD) curves of both materials at a current density of 100 mA/g, in which both materials displayed well overlapped GCD curves with a voltage of approximately 1.2 V. Additionally, FePcF_16_–rGO exhibited a higher capacity retention of 88.3% compared to FePc–rGO, which had 78.1%.

**Figure 4:**
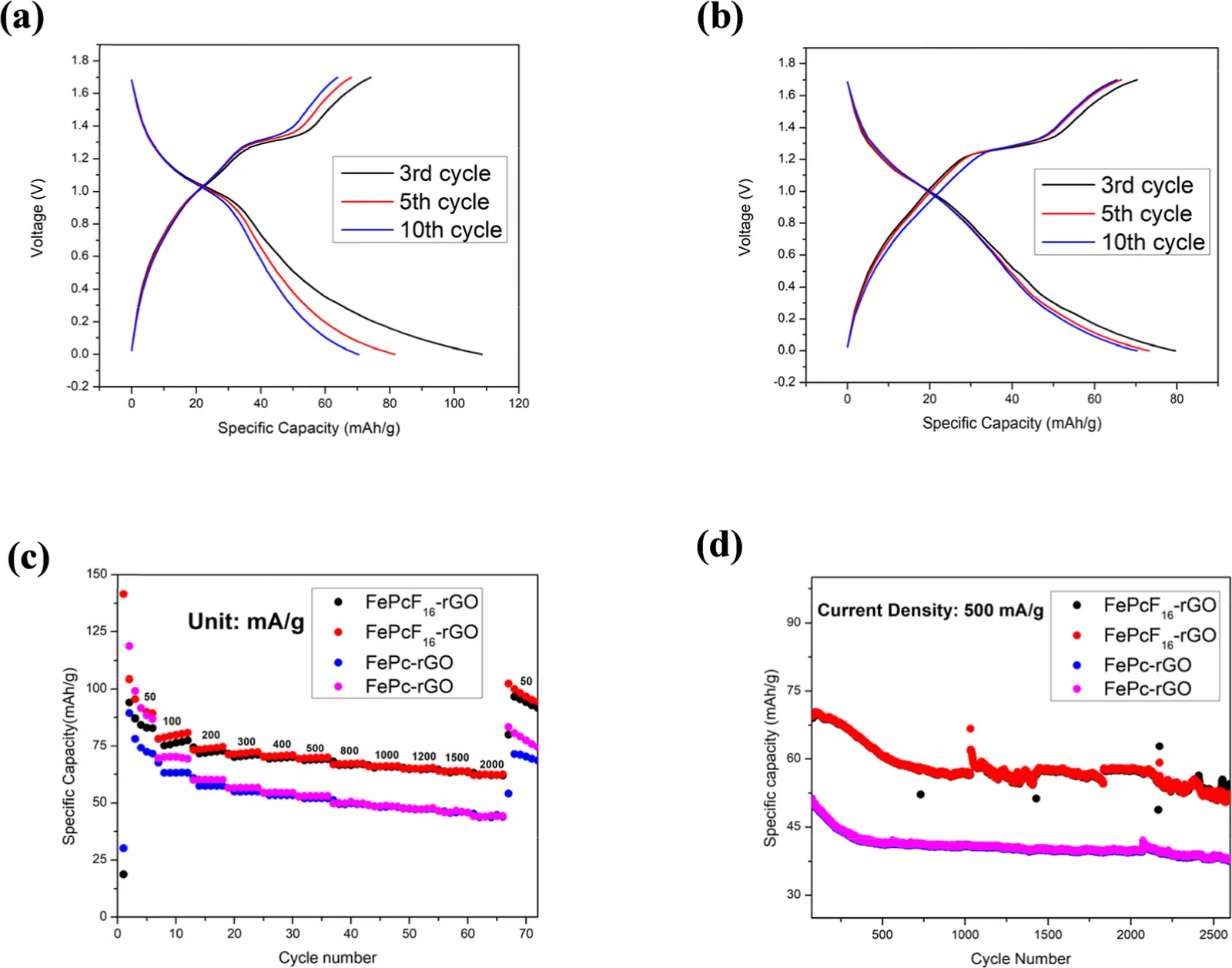
Electrochemical characterization of the prepared hybrids. (a) GCD curve of FePc–rGO at a current density of 100 mA/g. (b) GCD curve of FePcF_16_–rGO at a current density of 100 mA/g. (c) Rate performance of FePc–rGO and FePcF_16_–rGO hybrids. (d) Cyclic performance of FePc–rGO and FePcF_16_–rGO hybrids at a current density of 500 mA/g.

To further study their electrochemical performance, their rate performance was evaluated at 50, 100, 200, 300, 400, 500, 800, 1000, 1200, 1500, and 2000 mA/g, as shown in Figure 4c. Both materials showed promising rate capabilities up to 2000 mA/g. In the case of FePc–rGO, the discharge capacities were 78 mAh/g, 70 mAh/g, 59 mAh/g, 56 mAh/g, 54 mAh/g, 53 mAh/g, 49 mAh/g, 48 mAh/g, 47 mAh/g, 46 mAh/g, and 43 mAh/g, respectively.

Once again, FePcF_16_–rGO displayed improved performance with capacities of 96 mAh/g, 78 mAh/g, 73 mAh/g, 71 mAh/g, 70 mAh/g, 69 mAh/g, 67 mAh/g, 66 mAh/g, 65 mAh/g, 64 mAh/g, and 62 mAh/g, respectively. This improved rate capability demonstrates the positive effect of substituting hydrogen atoms with fluorine on battery performance.

Furthermore, Figure 4d demonstrates the long-term cyclic performance of both materials, in which FePc–rGO and FePcF_16_–rGO maintained capacities of 38 mAh/g and 52 mAh/g after 2500 cycles at a current density of 500 mA/g, which represents a promising capacity retention of 76 and 73%, respectively, and an average Coulombic efficiency of 98%. This is a notable improvement compared to pristine FePc, which exhibited a capacity retention of 56.7% after only 500 cycles under the same conditions.^29^ XRD was performed after cycling (Figure S8), and the FePcF_16_–rGO cathode maintained the characteristic phthalocyanine peaks, however, with less intensity showing a decrease in crystallinity after cycling.

### Reaction Kinetics and Charge Storage

To better understand the battery reaction kinetics, CV was performed at different scan rates. Figure 5a,b show the CVs of FePc–rGO and FePcF_16_–rGO, respectively, and both have one redox pair at 1.36 and 1.00 V, which can be attributed to the Fe^3+^/Fe^2+^ redox Pair.^39^ Further information about the charge storage mechanism can be obtained by utilizing the relationship between current (*i*) and scan rate (*v*), where a and *b* are empirical constants.

1 $$i=av^{b}$$

**Figure 5:**
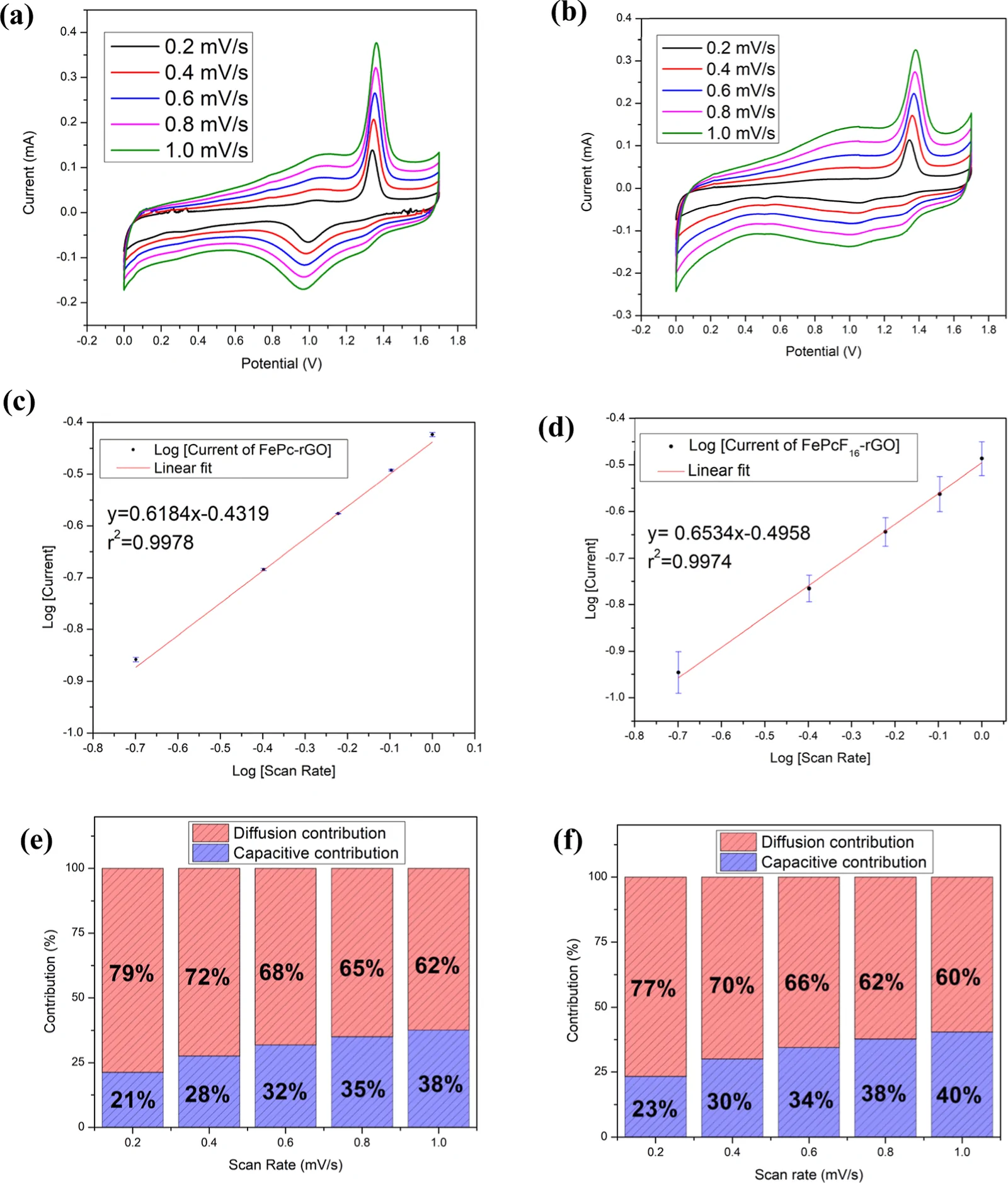
Kinetic study of FePc–rGO and FePcF_16_–rGO hybrids. (a) CV of FePc–rGO at different scan rates. (b) CV of FePcF_16_–rGO at different scan rates. (c) Linear fitting of log­[scan] and log­[current] of FePc–rGO. (d) Linear fitting log­[scan] and log­[current] of FePcF_16_–rGO. (e) Capacitive and diffusion charge storage contribution for FePc–rGO. (f) Capacitive and diffusion charge storage contribution for FePcF_16_–rGO.

By plotting the relation between the log of current as a function of the log of the scan rate, the contribution of diffusion-controlled processes and capacitive processes can be determined, in which a value for *b* close to 0.5 suggests that charge storage is predominantly via ion insertion and a value close to 1.0 suggests capacitive controlled charge storage.^40^

Figure 5c,d illustrate the linear relation between the logarithm of the peak current and the logarithm of the scan rate for FePc–rGO and FePcF_16_–rGO hybrids; in the case of FePc–rGO, the value of *b* is 0.61, and for FePcF_16_–rGO it was 0.65, which implies that while the charge is predominantly stored via an ion insertion mechanism, there is a significant contribution of surface-controlled processes.^41^ It is important to note that Figure 5d features a higher uncertainty due to the broader peaks associated with the redox process compared to Figure 5c, which had sharp redox peaks.

The contribution from each process can be estimated by applying the following formula

2 $$i_{v}=k_{1}v+k_{2}v^{1/2}$$

where *k*_1_ and *k*_2_ are constants associated with the nonfaradaic current and faradaic current, respectively.^38^ Rearranging this equation as follows allows the determination of the specific contributions of diffusion and capacitive processes.^42^

3 $$\frac{i}{v^{1/2}}=k_{1}v^{1/2}+k_{2}$$

Both hybrids showed a predominantly diffusion-controlled contribution, as illustrated in Figure 5e,f, which is expected given the *b* values of 0.61 and 0.65. Additionally, the significant capacitive contribution, from 21 to 38% at 0.2 mV/s and 1.0 mV/s for FePc–rGO and from 23 to 40% for FePcF_16_–rGO, indicates that the hybrid materials have pseudocapacitive behavior, which differs from that of pristine MPc, which is ion diffusion-controlled.^29^ This pseudocapacitance explains the improved rate performance since pseudocapacitance relies on surface redox reactions, as opposed to diffusion-controlled ion intercalation.^43,44^ Once again, FePcF_16_–rGO had more pseudocapacitive behavior compared with FePc–rGO, which further demonstrates the benefit of utilizing substituted MPcs as cathodes compared to unsubstituted MPcs.

Ex situ XRD (Figure S11) was performed to further gain insight into the ion storage mechanism. After discharging, there is an increase of intensity in the diffraction peaks of 9.7°, 11.3° which can be attributed to the intercalation of zinc into the structure. Additionally, new diffraction peaks appear at 33.7° and 58.7°. Furthermore, EDS (Figure S12 and Table S3) of the cathode after discharging shows the presence of zinc in the structure, 19.5% in weight contribution as well as chlorine corresponding to 4.42%. Based on the surface elemental analysis, the amount of Zn is greater than that of Cl, which indicates that Zn^2+^ is the main ion stored. However, the presence of chlorine in the EDS and the new diffraction peak at 33.7° suggests the formation of Simonkolleite as a byproduct during discharge.

Finally, electrochemical impedance spectroscopy (Figures S13 and S14) was used to determine the charge transfer resistance for each material, and FePc–rGO had a charge transfer resistance of 4.77 Ω, and FePcF_16_–rGO had a value of 5.07 Ω. The low values suggest fast kinetics, which further explains the high rate performance of the hybrids.

Given these results, we can establish that incorporating rGO and MPcs to prepare a hybrid material leads to increased battery performance. Additionally, substituting hydrogen atoms with fluorine increased the battery’s capacity. Further research will be conducted to evaluate the effects of different substituents on the phthalocyanine ring on overall battery performance and to elucidate the relationship between the phthalocyanine structure and specific capacity.

## Conclusions

In this work, FePc–rGO and FePcF_16_–rGO hybrids were prepared, and their electrochemical properties were evaluated in aqueous zinc batteries, in which incorporating rGO and FePc by π–π interactions into a hybrid material led to an increase in battery performance compared to pristine FePc. The FePc–rGO hybrid maintained a capacity of 38 mAh/g after 2500 cycles, with a capacity retention of 76% and a high-rate capability of 2000 mA/g. Furthermore, incorporating fluorine atoms into the phthalocyanine structure resulted in improved specific capacity compared to the unsubstituted FePc–rGO cathode. The FePcF_16_–rGO hybrid displayed a capacity of 52 mAh/g after 2500 cycles, a capacity retention of 73%, and a high-rate capability of 2000 mA/g. CV determined the pseudocapacitive behavior of both FePc–rGO and FePcF_16_–rGO hybrids, with a contribution of over 20% to the overall charge storage.

CV determined that both hybrids feature pseudocapacitive behavior with over a 20% contribution to the overall charge storage, which explains why the prepared materials feature a high rate capability. This work demonstrates the importance of the substituents of the phthalocyanine ring in improving the battery performance of phthalocyanine-based cathodes for the design of high-performance aqueous zinc batteries.

## Supplementary Material



## Notes

Additional FT-IR, 19F NMR, XRD, EDS, and elemental composition data, along with GCD curves and electrochemical impedance spectroscopy results (PDF)

## Abbreviations

MPc: metal phthalocyanine
rGO: reduced graphene oxide
Pn: phthalonitrile
