# Supplementary material for: Fluorinated Iron Phthalocyanine–rGO Hybrid Cathodes for Fast and Stable Aqueous Zinc Batteries
Source: ACS Omega. 2026 Aug 24;11(35):53031–9. doi: 10.1021/acsomega.6c05388 (PMC13563999; doi:10.1021/acsomega.6c05388)
Supplement: Supplementary file 1 [file ao6c05388_si_001.pdf]

Fluorinated Iron Phthalocyanine-rGO Hybrid Cathodes for Fast and Stable Aqueous Zinc Batteries

Andres E. Araujo-Correa[1], Yamna D. Acosta-Tejada[2], Lexis N. Feliz-Hernandez[2], Wentao Hou[1], Laura Catalina Cruz[1], Lisandro Cunci[1], Karilys González-Nieves[2], Xianyong Wu[1*], Dalice M. Piñero Cruz[1*].*

Affiliation: [1]University of Puerto Rico-Rio Piedras. [2]University of Puerto Rico-Carolina.

Supplemental Information

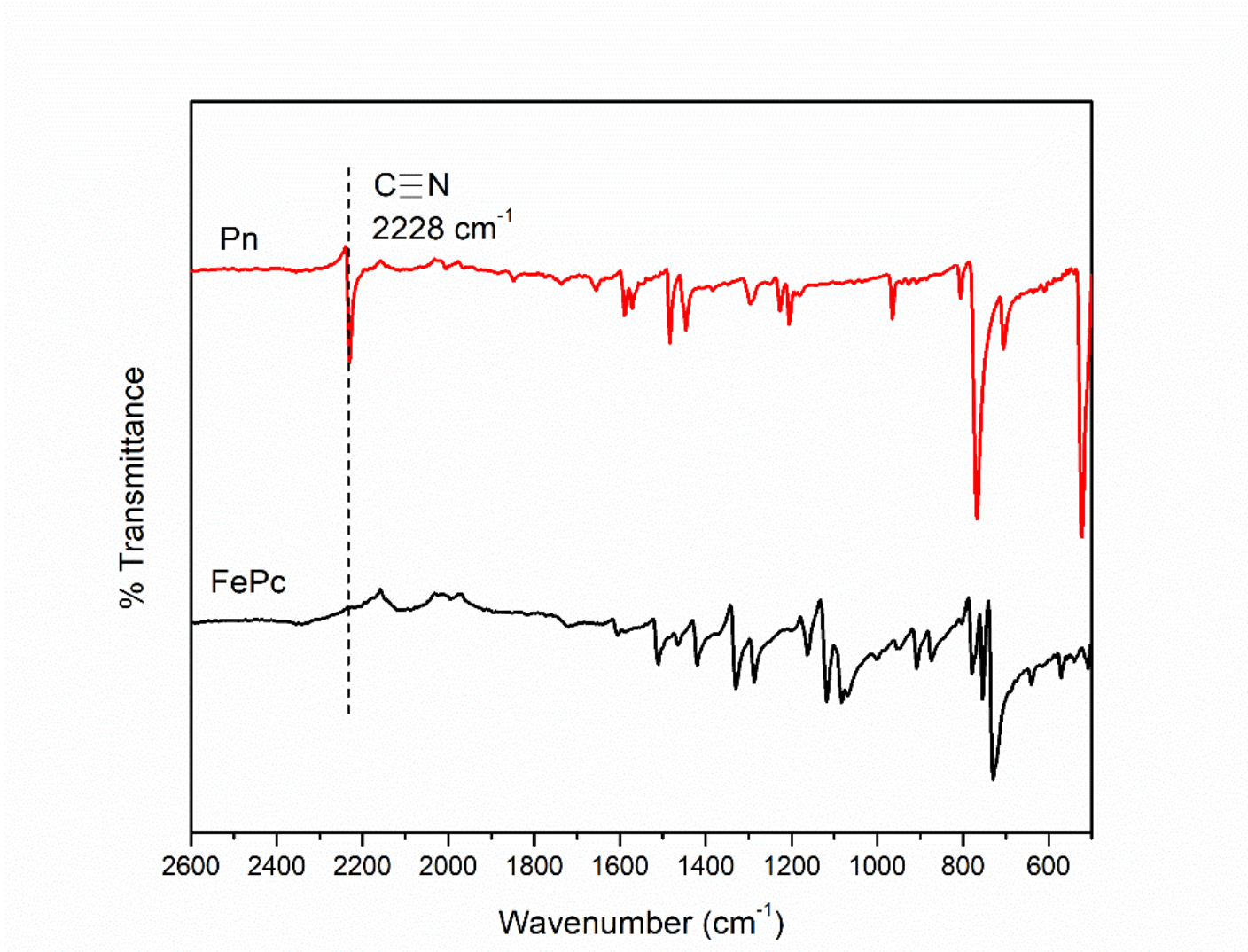

Figure S1. FT-IR of FePc and phthalonitrile precursor.

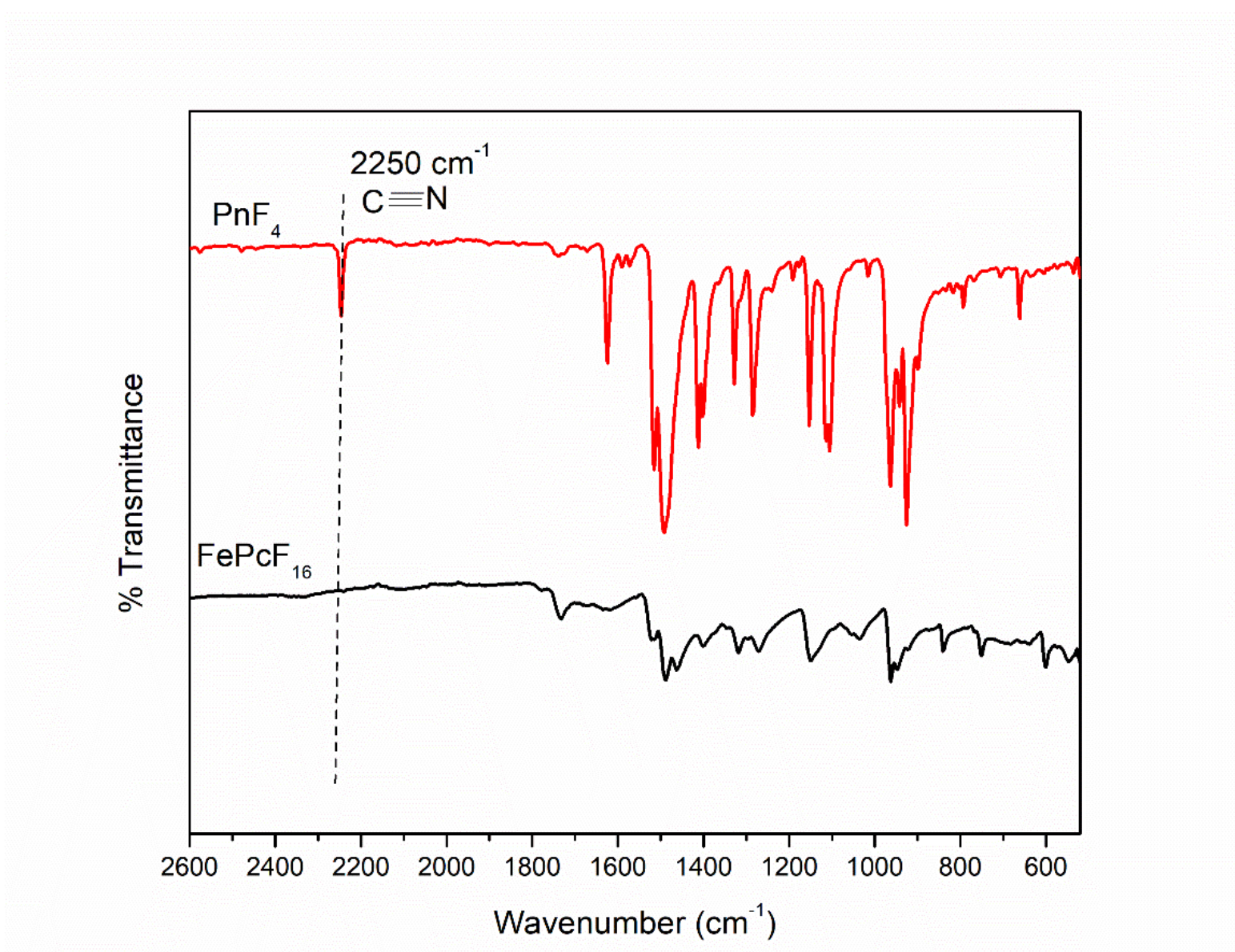

Figure S2. FT-IR of $FePcF_{16}$ and tetrafluoro phthalonitrile precursor

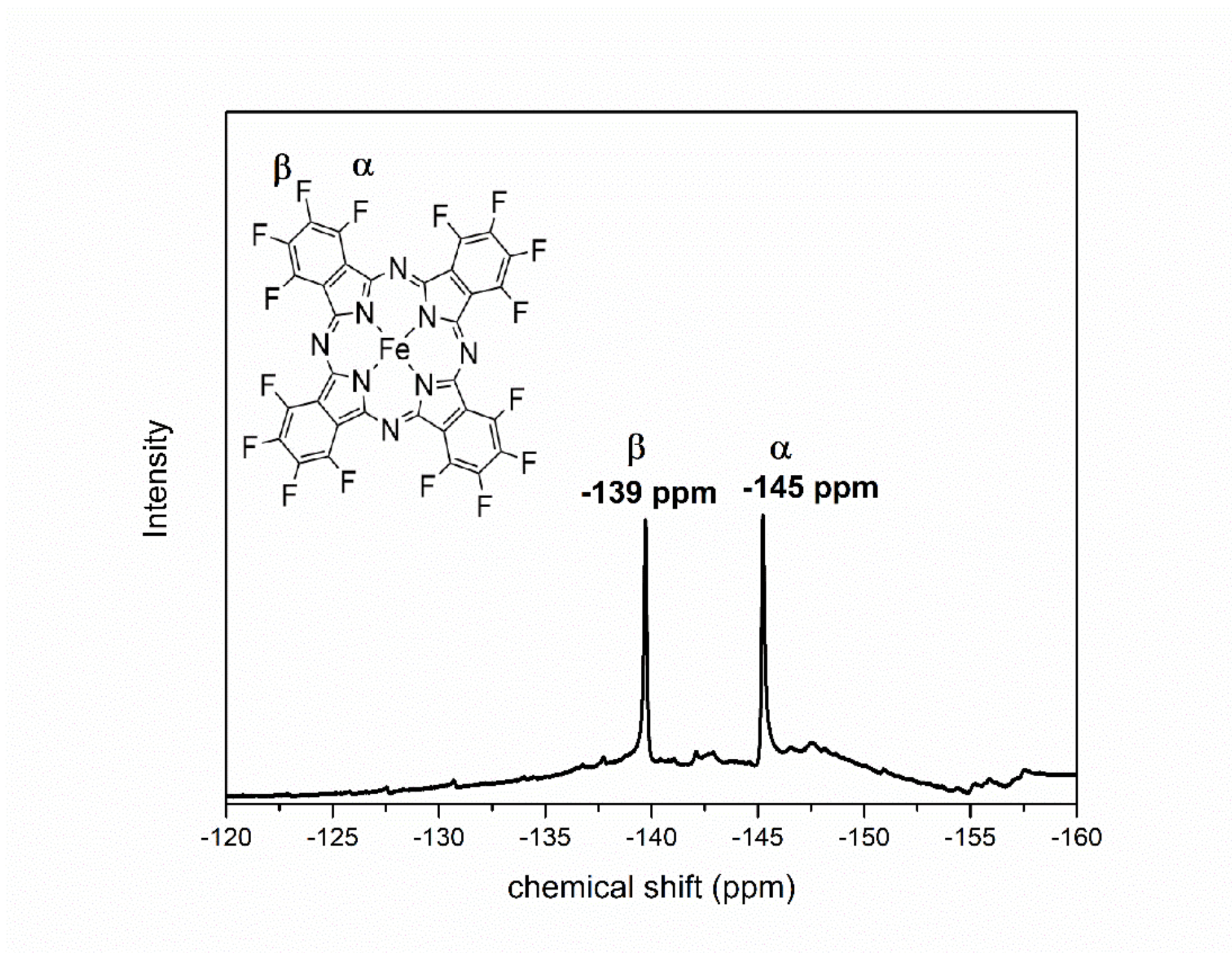

Figure S3. ^{19}F-NMR of $FePcF_{16}$ in DMSO-d6.

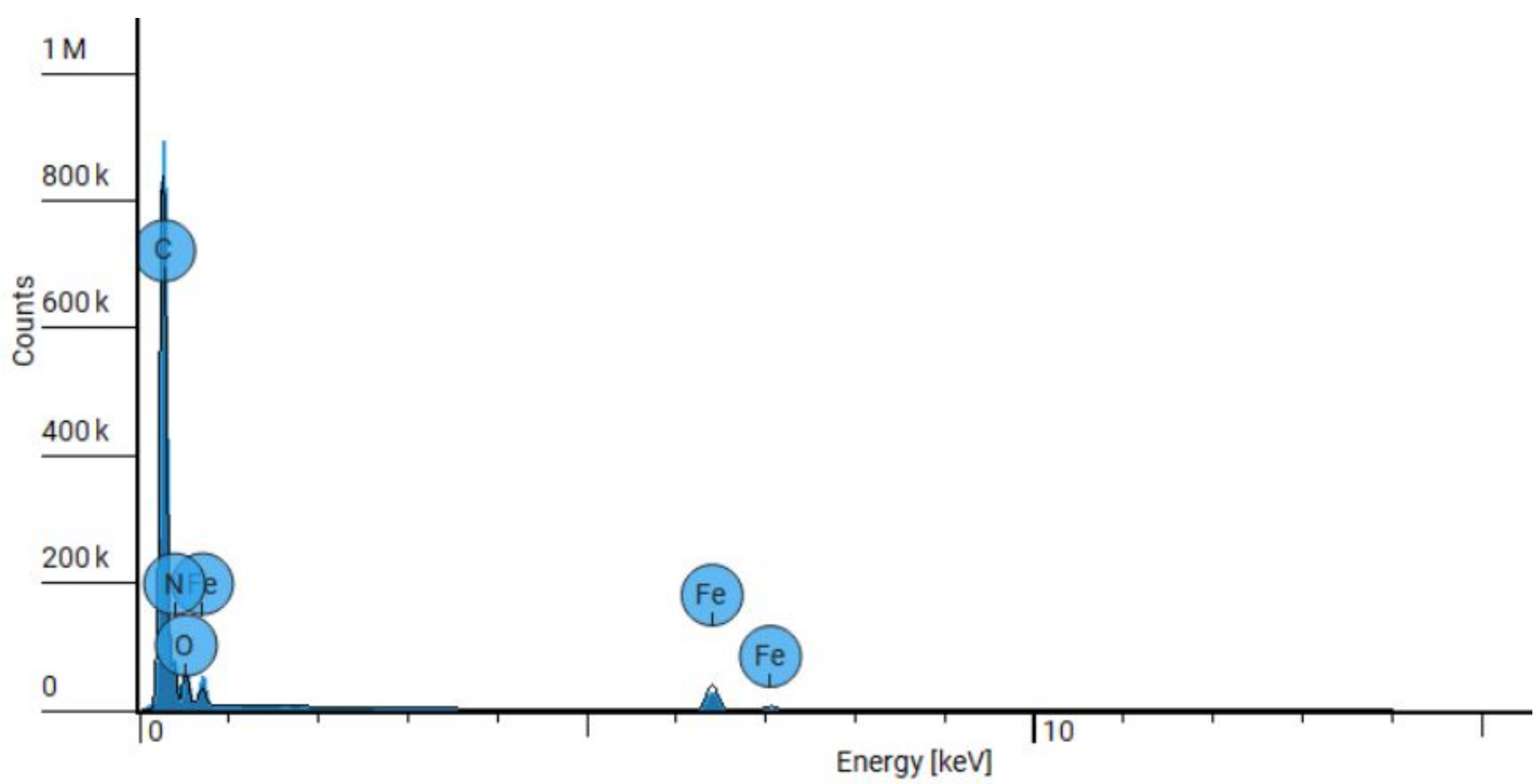

Figure S4. EDS of FePc-rGO hybrid.

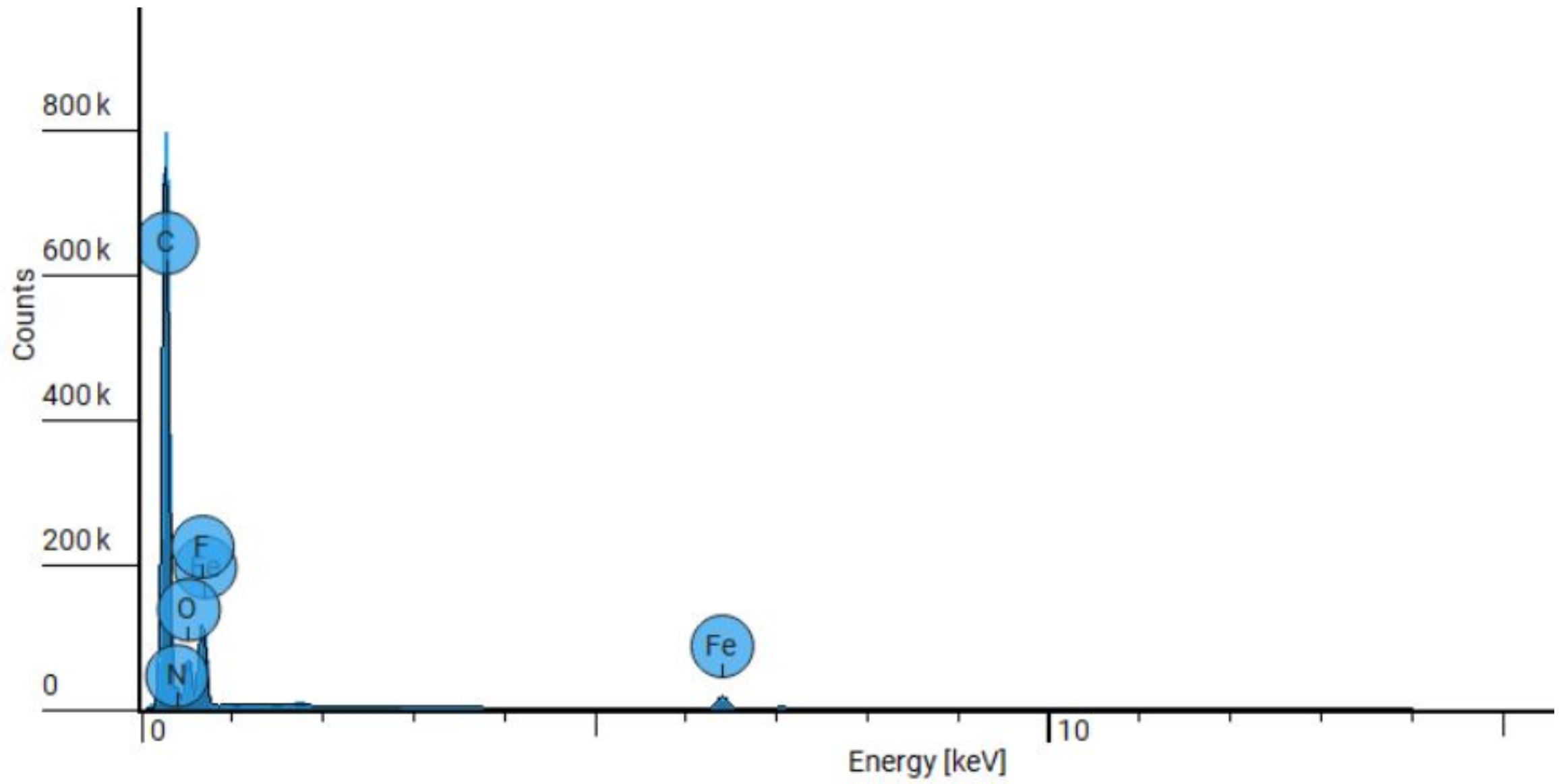

Figure S5. EDS of $FePcF_{16}$-rGO hybrid.

	Element Number	Element Symbol	Element Name	Atomic Conc. [%]	Weight Conc. [%]
	6	C	Carbon	64.96	53.85
	7	N	Nitrogen	20.46	19.78
	8	O	Oxygen	10.86	11.99
	26	Fe	Iron	3.73	14.39

Table S1. Elemental composition of FePc-rGO hybrid.

	Element Number	Element Symbol	Element Name	Atomic Conc. [%]	Weight Conc. [%]
	6	C	Carbon	65.94	55.75
	7	N	Nitrogen	9.61	9.48

	**8**	**O**	**Oxygen**	**12.17**	**13.71**
	**9**	**F**	**Fluorine**	**10.48**	**14.01**
	**26**	**Fe**	**Iron**	**1.80**	**7.06**

Table S2. Elemental composition of FePcF16-rGO hybrid.

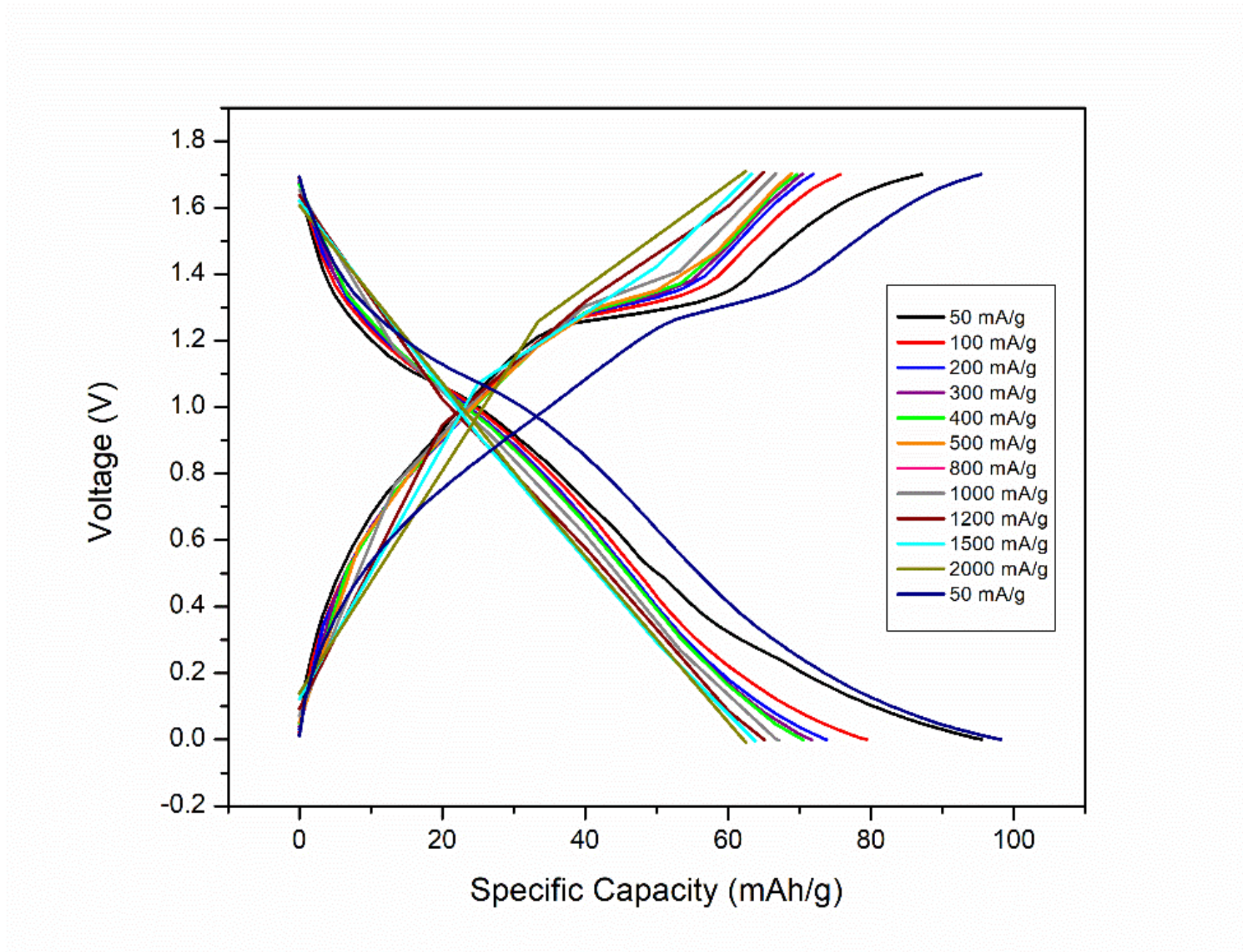

Figure S6. GCD curve of FePc-rGO rate performance at different current densities.

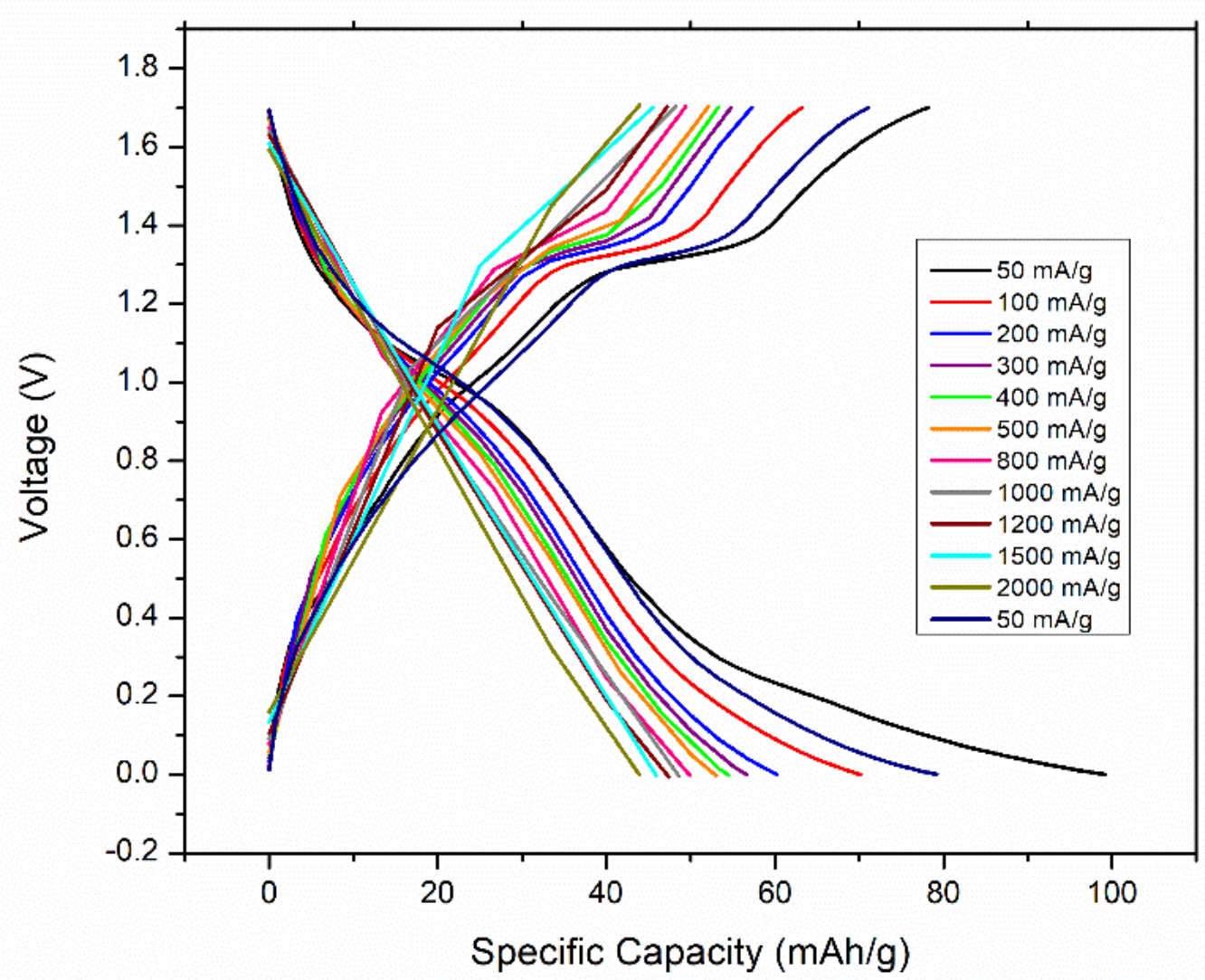

Figure S7. GCD curve of FePcF16-rGO rate performance at different current densities.

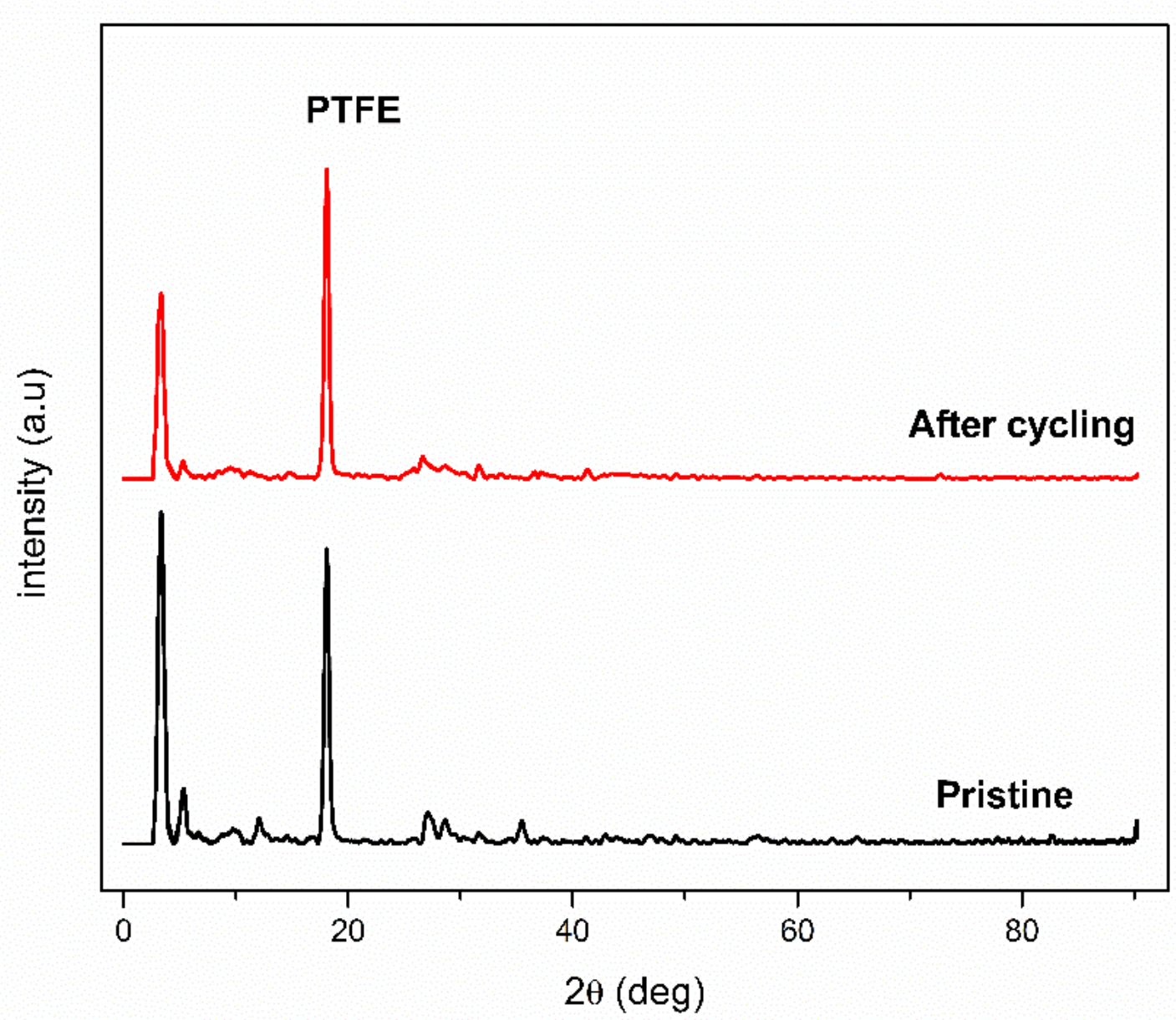

Figure S8. XRD of FePcF16-rGO cathode before and after cycling.

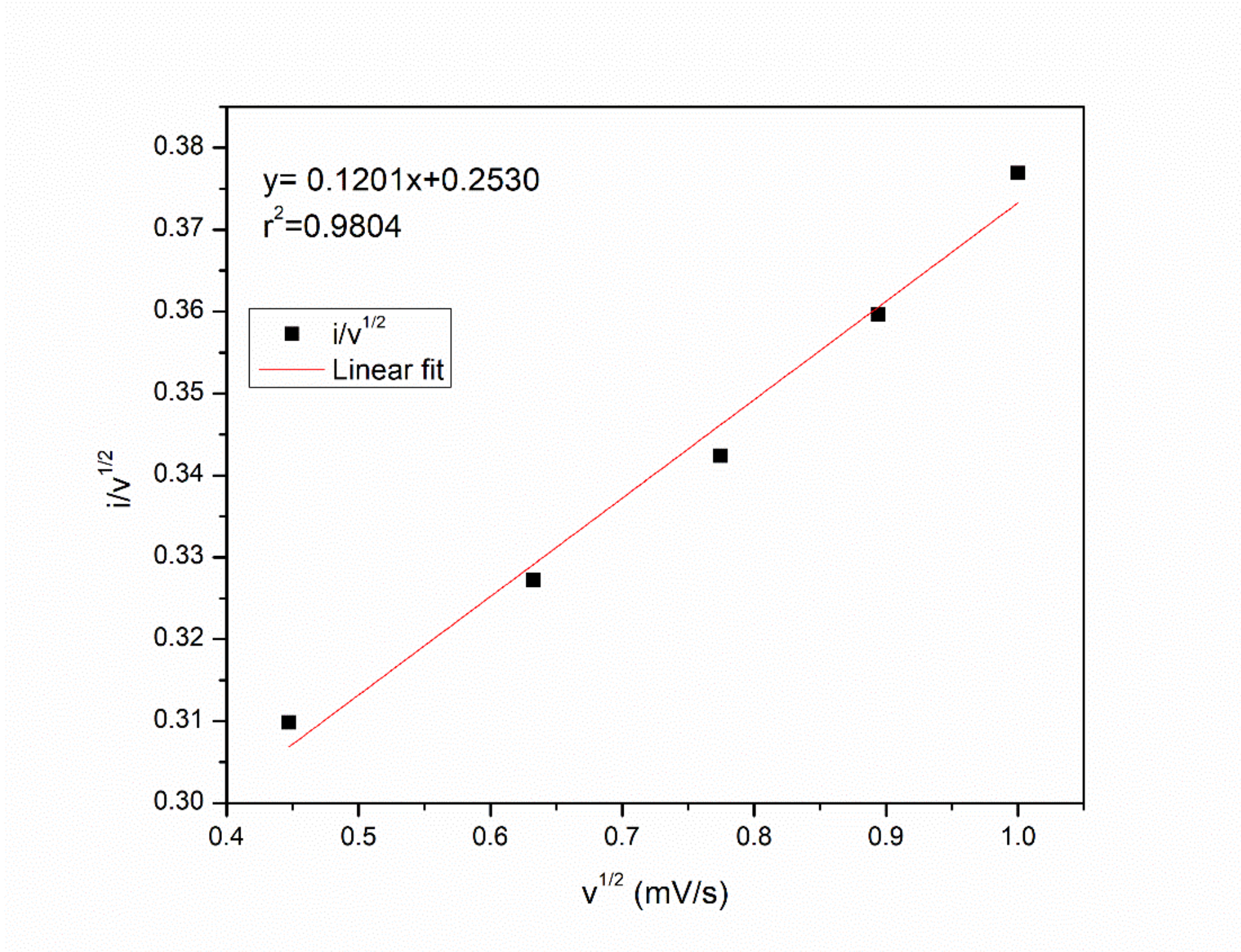

Figure S9. Linear Fit of $i/v^{1/2}$ vs $v^{1/2}$ of FePc-rGO hybrid.

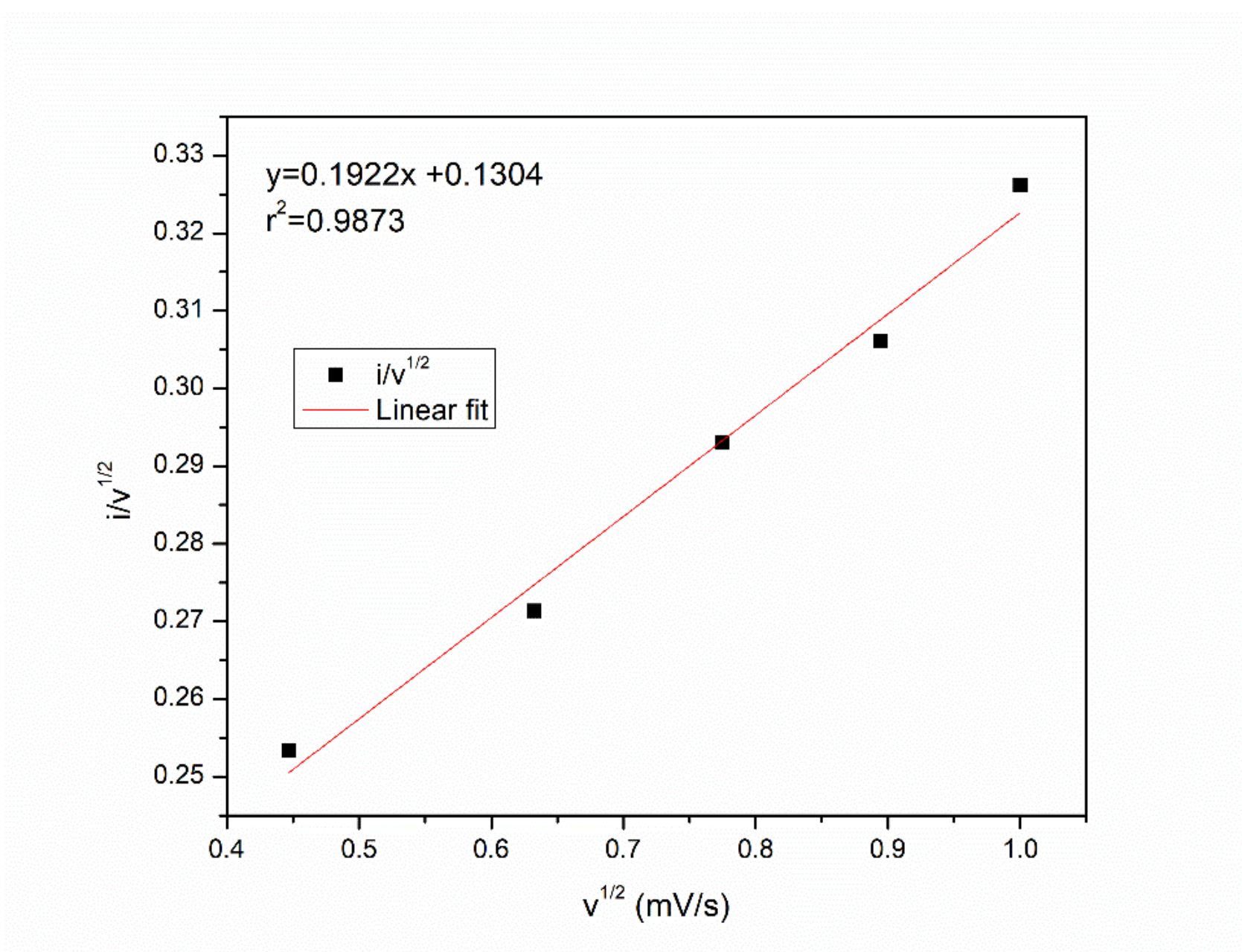

Figure S10. Linear Fit of $i/v^{1/2}$ vs $v^{1/2}$ of $FePcF_{16}$-rGO hybrid.

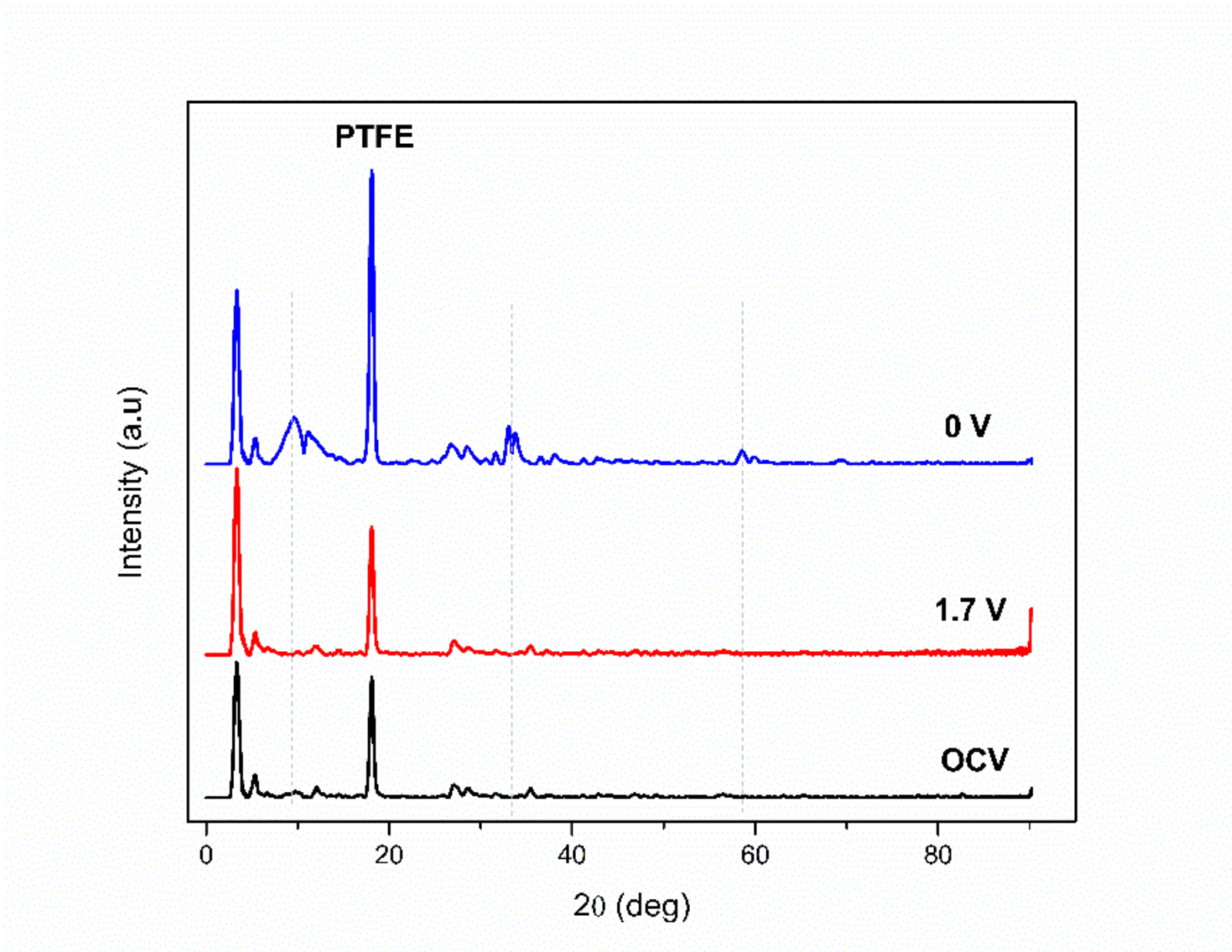

Figure S11. Ex-situ XRD of $FePcF_{16}$-rGO cathode.

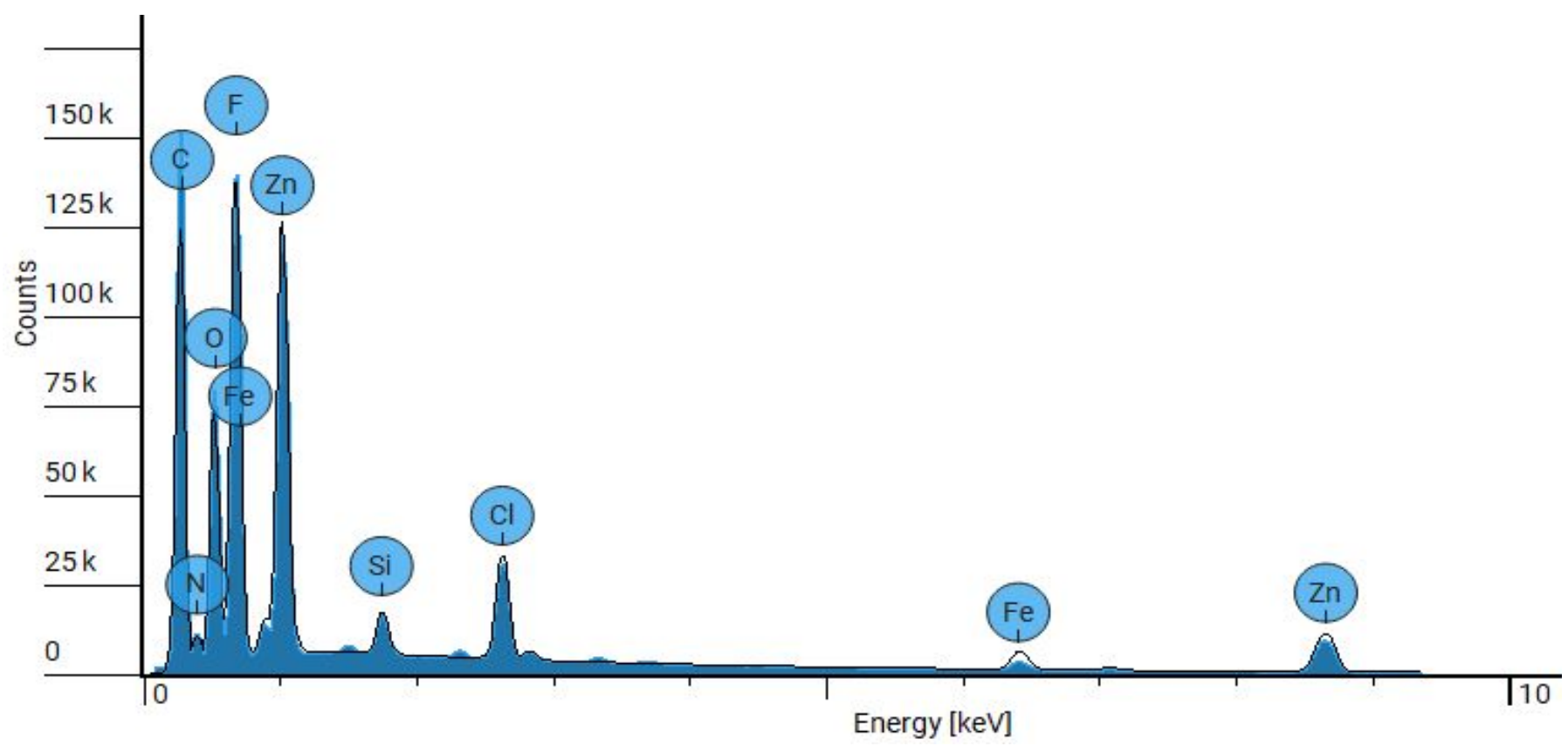

Figure S12. EDS of $FePcF_{16}$-rGO cathode after discharge.

	Element Number	Element Symbol	Element Name	Atomic Conc. [%]	Weight Conc. [%]
	6	C	Carbon	49.62	32.76
	7	N	Nitrogen	3.91	3.02
	8	O	Oxygen	17.02	14.97
	9	F	Fluorine	19.92	20.80
	14	Si	Silicon	0.72	1.11
	17	Cl	Chlorine	2.27	4.42
	26	Fe	Iron	1.11	3.42
	30	Zn	Zinc	5.42	19.50

Table S3. Elemental composition of $FePcF_{16}$-rGO hybrid after discharge.

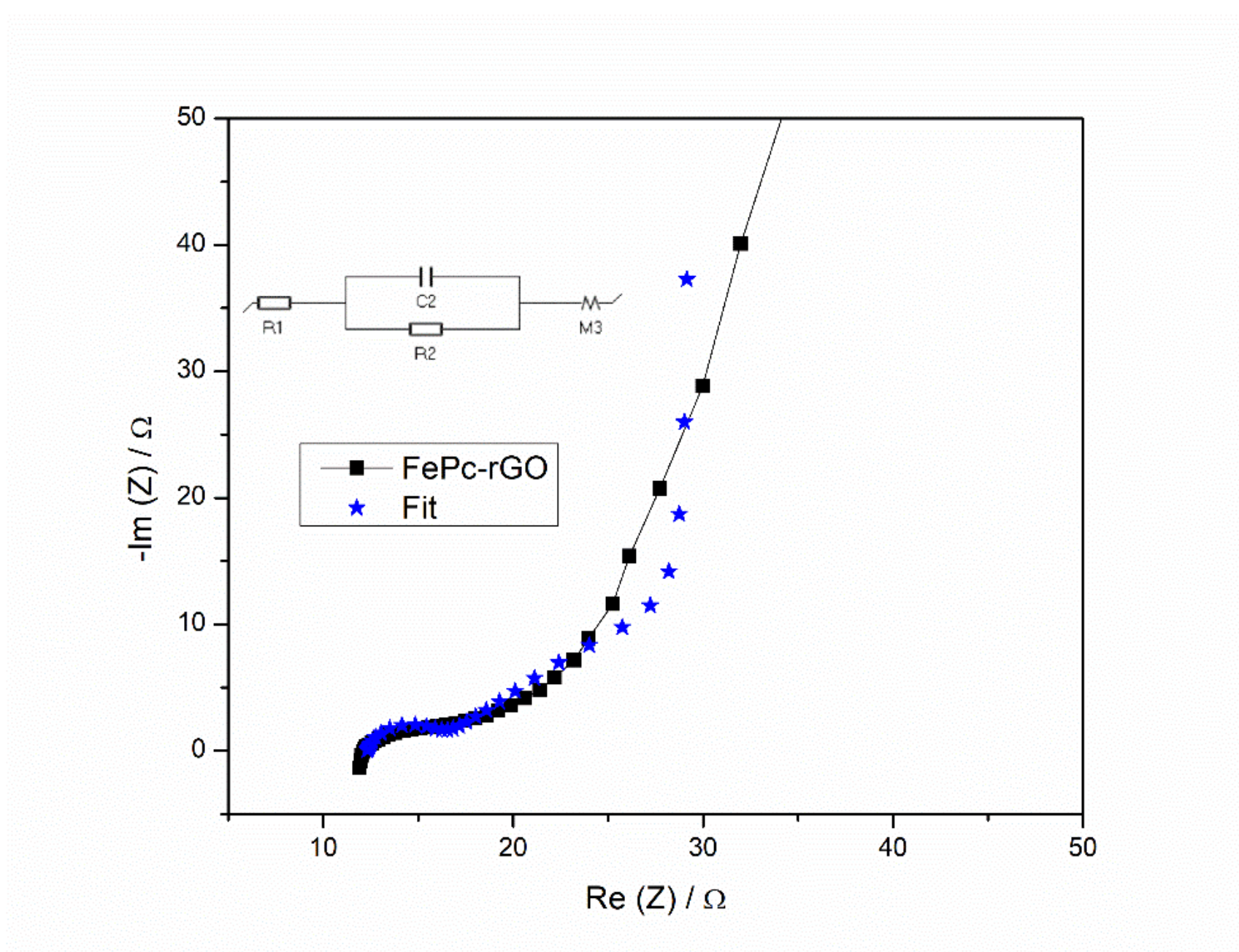

Figure S13. Electrochemical Impedance Spectroscopy of FePc-rGO and simulated circuit.

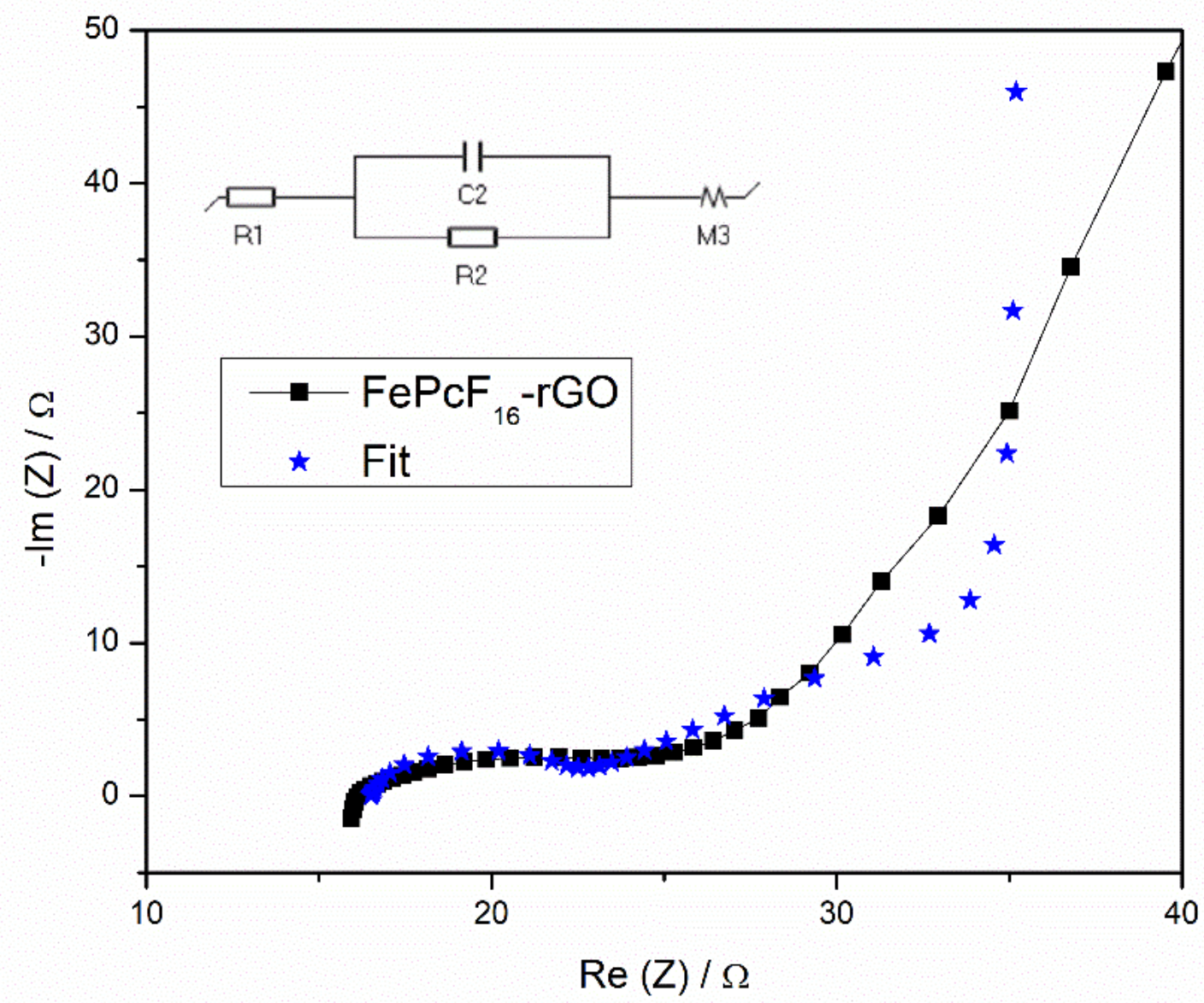

Figure S14. Electrochemical Impedance Spectroscopy of $FePcF_{16}$-rGO and simulated circuit.
